# A New Work on Diseases of the Chest

**Published:** 1855-10

**Authors:** 


### A NEW WORK ON DISEASES OF THE CHEST.

Prof. Flint, as we have stated in a former number, is engaged in preparing for the press an elaborate work on Diseases of the Chest. We learn that the work is far advanced, a large portion of it having been printed, and that it will be completed in a few months. Prof. Flint's time having been so much engrossed by this undertaking, he has been obliged to suspend, temporarily, the publication of his Lectures on Diseases of the Skin. His work being now nearly off his hands, he will soon have leisure to return to his Lectures.

